# Mitochondria in Health and Disease

**DOI:** 10.1155/2014/814042

**Published:** 2014-03-03

**Authors:** José Magalhães, Paola Venditti, Peter J. Adhihetty, Jon Jay Ramsey, António Ascensão

**Affiliations:** ^1^Department of Sport Biology, Research Centre in Physical Activity, Health and Leisure (CIAFEL), Faculty of Sport, University of Porto, Rua Dr. Plácido Costa 91, 4200-450 Porto, Portugal; ^2^Sezione di Fisiologia, Dipartimento delle Scienze Biologiche, Università degli Studi di Napoli “Federico II”, Via Mezzocannone 8, 80134 Napoli, Italy; ^3^Department of Applied Physiology and Kinesiology, College of Health and Human Performance, University of Florida, P.O. Box 118205, Gainesville, FL 32611, USA; ^4^VM Molecular Biosciences, University of California, Davis, CA 95616, USA

Mitochondria are dynamic and complex cellular organelles that are involved in a wide range of cellular events and are essential for tissue adaptation, survival, death, and renewal. In addition to their important role in energy metabolism making them popularly known as the cellular powerhouse in textbook definitions, mitochondria are malleable structures that are also intimately involved in controlling cellular redox status, cellular signaling, calcium homeostasis, and cell death and autophagy processes. Thus, mitochondria have emerged from simply being the powerhouse of the cell to being at the forefront of numerous research avenues. In fact, mitochondrial perturbations evoked by physiological and pathological stimuli have been shown to contribute towards the pathogenesis of many diseases and mitochondrial research now constitutes a very significant and ever-expanding research area. It is now understood that mitochondria and their associated pathways may represent areas for the development of preventive and therapeutic strategies to potentially mitigate diseases/disorders such as diabetes, obesity, neurodegeneration, and sarcopenia.

In the present issue of Oxidative Medicine and Cellular Longevity devoted to mitochondria in health and disease, a variety of original research articles were published covering distinct aspects of cellular physiology and adaptation involving mitochondria. These include the role of mitochondrial and peroxisome group VIB phospholipase A2 (iPLA2g) in *β*-cell proliferation and redox control (Bao et al., 2013), the study of mitochondrial metabolic and structural phenotypes in liver and skeletal muscle from obese animals (Cao et al., 2013), the modulator effects of hydrogen disulfide in neuronal cells and mitochondria (Guo et al., 2013), the mechanisms by which astragaloside IV protects against oxidative stress-induced increased permeability transition pore opening in cardiac cell line (He et al., 2012), the effects of alpha-lipoic acid on mitochondrial superoxide and glucocorticoid-induced hypertension (Ong et al., 2013), and the development of in vitro approaches to study population mitochondrial genomic variations (Lin et al., 2013). In the present issue, the potential use of two-photon microscopy probes for the study of mitochondrial redox environment (Kim and Cho, 2013) and the potential of translocator protein 18 as therapeutic target and also a diagnostic tool for cardiovascular diseases (Qi et al., 2013) are also reviewed and discussed. It is our belief that the articles in this special mitochondrial issue could provide an important contribution to improve the use of mitochondrial-related models for health and disease research, as well as identifying mitochondrial pathways and associated mechanisms as important subcellular targets in the prevention and treatment of many pathological conditions.

*José  Magalhães* *José  Magalhães* *Paola Venditti* *Paola Venditti* *Peter J. Adhihetty* *Peter J. Adhihetty* *Jon Jay Ramsey* *Jon Jay Ramsey* *António Ascensão* *António Ascensão*

